# A Spellbinding Interplay Between Biological Barcoding and Nanotechnology

**DOI:** 10.3389/fbioe.2020.00883

**Published:** 2020-09-08

**Authors:** Shehla Munir, Sarfraz Ahmed, Muhammad Ibrahim, Muhammad Khalid, Suvash Chandra Ojha

**Affiliations:** ^1^Department of Biochemistry, Bahauddin Zakariya University, Multan, Pakistan; ^2^Department of Basic Sciences, University of Veterinary and Animal Sciences, Lahore, Pakistan; ^3^Department of Chemistry, Khwaja Fareed University of Engineering & Information Technology, Rahim Yar Khan, Pakistan; ^4^Department of Infectious Diseases, The Affiliated Hospital of Southwest Medical University, Luzhou, China

**Keywords:** nano-barcoding, nano-biosensors, biobarcoding, barcoded nanoparticles, nanotechnology, cytokines, proteins, nucleic acids

## Abstract

Great scientific research with improved potential in probing biological locales has remained a giant stride. The use of bio-barcodes with the potential use of nanotechnology is a hallmark being developed among recent advanced techniques. Biobarcoding is a novel method used for screening biomolecules to identify and divulge ragbag biodiversity. It establishes successful barcoding projects in the field of nanomedical technology for massively testing disease diagnosis and treatment. Biobarcoding and nanotechnology are recently developed technologies that provide unique opportunities and challenges for multiplex detection such as DNAs, proteins and nucleic acids of animals, plants, viruses, and various other species. These technologies also clump drug delivery, gene delivery, and DNA sequencing. Bio-barcode amplification assay (BCA) is used at large for the detection and identification of proteins and DNAs. DNA barcoding combined with nanotechnology has been proven highly sensitive rendering fast uniplex and multiplex detection of pathogens in food, blood, and other specimens. This review takes a panoramic view of current advances in nano bio-barcodes which have been summarized to explore additional applications such as detection of cytokines, neurotransmitters, cancer markers, prostate-specific antigens, and allergens. In the future, it will also be possible to detect some fungi, algae, protozoa, and other pollutants in food, agriculture, and clinical samples. Using these technologies, specific and efficient sensors would possibly be developed that can perform swift detections of antigens, allergens, and other specimens.

## Introduction

Many scientific and industrial sectors can be significantly improved and revolutionized by the new science of nanotechnology (Lyle et al., 2015). Two main factors have proved nanotechnology as a powerful engine of innovations: (1) industrial interest in nano-engineered materials. It leads to the development of many innovations like chemically active or inert additives (that impart beneficial qualities to consumer products such as increased surface area or hardness), coloring, ultraviolet protection, and antimicrobial agents; (2) interest to improve human-environmental interaction, especially concerns about the harmful effects on environment and humans (Stark et al., 2015). With time, nanotechnology emerged as an integral field due to its extensive applications in information technology and telecommunication, physics, chemistry, and life sciences (Ochs et al., 2006). It is also used in effective drug delivery and gene delivery to make liposomes in cardiac therapy and dental care (Ochs et al., 2006). Its vast applications are in molecular fields like structural DNA nanotechnology for assembling barcodes like nucleic acid barcodes (Wang P. et al., 2014). DNA sequencing performed through nanopores can also help in the diagnosis of infectious and cancerous diseases (Wanunu, 2012; Grodzinski et al., 2014). In recent years, several research studies have been conducted for developing nanotechnology-based DNA barcoding and biobarcoding (Wanunu, 2012; Grodzinski et al., 2014). Currently, recent challenges in research studies are to explore marvelous applications of nanotechnology with the potential use of DNA barcoding and biobarcoding.

Bio-barcode is a bioinformatics platform to be utilized as a coding system using nanotechnology. Biologists built a DNA barcode server and database using a biobarcoding system. It facilitates the compilation, depository, analysis, and publication of high-quality barcoded DNA records (Goluch et al., 2009). Bio-barcode provides the tools that launch successful barcoding projects in biodiversity research (Lim et al., 2009). For clinical purposes, Bio-barcode Assay (BCA) is used for the detection and specification of proteins and target DNA, which may have been proved to be pathogenic (Goluch et al., 2009). At an elaboration level, BCA also provides Polymerase Chain Reaction (PCR)-like amplification for nucleic acid and protein without the use of enzymatic amplification (Goluch et al., 2009). In BCA (bio-barcoded amplification) based assays, barcoded DNA and nanoparticles are used that can detect trace proteins, cytokines, and some neurotransmitters (Thaxton et al., 2005, 2009). Barcoded nanotechnology is used for the detection and diagnosis of cancer markers, and other vast range of infectious antigens in blood, food, and clinical samples. It can also detect prostate-specific antigens (Bao et al., 2006) by barcode lateral flow immunoassay. A barcode lateral flow immunoassay is used to optimize antibody affinity for a specific antigen (Liu et al., 2015).

DNA is a central part of genetic material which is also used in nanoscale engineering (Peng et al., 2012). DNA has many unique properties like biocompatibility, nanoscale controllability (Roh et al., 2011), and the capacity to recognize subcellular molecules. Therefore, nanotechnology in combination with DNA barcodes is an innovative technique to diagnose infectious pathogens in food, blood, and clinical samples. DNA is an extraordinary multifaceted material for developing nanoarchitecture motifs. Its vast applications have evolved between physics, chemistry, biology, computer science, and material science, etc. DNA based nanotubes and nanogrids have been constructed (Lund et al., 2006). Similarly, DNA scaffolds have been used for developing nanoelectronics. Even, DNA based nanomechanical devices have been designed (Lund et al., 2006). To identify the species, standard regions of the DNAs are sequenced in the form of a barcode. Since its origin back in 2003, DNA barcoding has been emerged as a novel tool and helped scientists to identify millions of species. Taxonomic classification consumes a lot of time to identify the species based on morphology (Coissac et al., 2016).

Approximately, 950 years are required to identify species worldwide using taxonomic methods (Rajpoot et al., 2016; Zinger and Philippe, 2016). DNA barcoding replaces the traditional taxonomic method to identify species as an innovative tool (Smith et al., 2008). In DNA barcoding, short genetic sequences in a DNA sample of animal, plant or any other species are used as markers for their identification and authentication (Hebert and Gregory, 2005). The universal barcode marker in animals is cytochrome c oxidase (CO1) that has been sliced from the mitochondrial genome, while in plants matK, ITS, rbcL, trnL-F, trnH-psbA, 5S-rRNA, and 18S-rRNA are specific barcode markers (Cowan and Fay, 2012). DNA barcoding is also applicable in the assessment of conservation impact, monitoring of biodiversity and illegal trades, and forensic analyses (Ferri et al., 2008; Ghosh et al., 2011; Nithaniyal et al., 2014). DNA barcoding has also been helpful to probe the *Mycobacterium tuberculosis*, and single–point mutation in exon21 of the epidermal growth factor receptor which implies lung cancer (Lebonah et al., 2014). It can also be used as a universal tool for food traceability to identify and quantify biological specimens. Thus, DNA barcoding using nanotechnology is being adopted as a novel tool to be used widely (Lebonah et al., 2014).

Use of nano-based detection has incremented the sensitivity up to ten folds as compared to the conventional methods of detection like radio-immunoassay, microarrays, enzyme-linked immunosorbent assay (ELISA), PCR, micro electrochemical biosensors, mass sensitive biosensors, and others (Tallury et al., 2010). However, there is a dire urge for diagnosing some fungi, algae, protozoa, and pollutants in food and agriculture using nano barcoding system (Tallury et al., 2010). In this study, we have summarized some applications developed by nanotechnology, biobarcoding, and with a major focus on DNA barcoding into the unified application so that all the features of these three technologies may view as a solo platform. This review would provide a panoramic overview of some recently developed applications of nanotechnology combined with DNA barcodes and a minor coverage of related bio-barcodes.

### Classification of Nanoparticles

Nanoparticles can be classified into various types according to the size, morphology, physical, and chemical properties. They have gained prominence in technological advancements due to their astounding physicochemical and biological properties including melting point, electrical and thermal conductivity, catalytic activity, light absorption, and scattering resulting in enhanced performance over their bulk counterparts. Based on physicochemical properties, they can be organized into: carbon-based nanoparticles, ceramic nanoparticles, metal nanoparticles, semiconductor nanoparticles, polymeric nanoparticles, and lipid-based nanoparticles (Khan et al., 2019). For detailed description on nanoparticles classification, readers are directed to these references (Jeevanandam et al., 2018; Khan et al., 2019). In recent years, nanoparticles have been used effectively in research areas, photocatalysis, photodegradation of dyes, diagnostics, detection and imaging of biomolecules, drug delivery to targeted sites, and in environmental and bio-analytical applications (Table 1). Some of the important ones used in biobarcoding include:

**TABLE 1:** General data on nanoparticle-based bio-barcode sensing.

Types of nanoparticles	Role of nanoparticles	Barcoding molecules	Specific targets	Detection techniques	Applications	References
Magnetic nano- particles and Quantum dot	Label	Barcoded oligonucleotide sequences	Bacterial DNA	Electrophoresis	Multiplex detection of *S. aureus*, MRSA, and *K. pneumoniae*	Nam et al., 2005
Gold nano- particles and Magnetic nano- particles	Capture probes	Barcoded oligonucleotide sequences	DNA	Fluorescence	Detection of Exotoxin A gene in *P. aeruginosa*	Amini et al., 2016
Gold nano- particles	Label and probe capture	Barcoded oligonucleotide sequences	HIV Capsid protein p24 antigen	Electrophoresis	Diagnosis of hepatitis	Dong et al., 2012
Magnetic nano- particles	Immunocomplex	–	PVX	Light scattering	Diagnosis of plant diseases	Panferov et al., 2016
Silver nano- particles	Label	Tumor penetrating peptide RPARPAR	PPC1 Prostate cancer cells	MS	Diagnosis of Prostate cancer	Fogal et al., 2008; Teesalu et al., 2009; Agemy et al., 2011
Silver nano- particles	Label	Tumor homing peptide GKPK	M21 melanoma cells	MS	Diagnosis of skin cancer	Fogal et al., 2008; Teesalu et al., 2009; Agemy et al., 2011
Gold nano- particles	Label	Single stranded thiol capped oligonucleotide sequences	Small molecules	Fluorescence	Development of optical biosensor for detection of very small molecules	Rees et al., 2016
Silver nano- particles	Transducer modifier	Barcoded dsDNA	DNA	Fluorescence	Detection of known piece of DNA	Zhou et al., 2014
Polystyrene nanoparticles	Immunocomplex	Bio-barcoded DNA	Target protein	Fluorescence	Detection of CRP in plasma	Broto et al., 2017
Silicon nano- particles	Label	Barcoded ssDNA	IL-2	Light absorption	Detection of IL-2	Nam et al., 2005
Gold nano-particles	Label	Bio-barcoded dsDNA	TAP	Fluorescence	Detection of small molecules e.g., TAP	Zhang et al., 2018
Gold nano- particles	Capture probe	Barcoded ssDNA	PSA	Light scattering	Detection of PSA	Bao et al., 2006
Silver enhancement gold nanoparticles	Scatter light	Thiol capped ssDNA	TAP	Light scattering	Detection of TAP	Zhang et al., 2018
Gold nanoparticles	Capture probe	Thiol capped dsDNA	Target protein	qPCR	Detection of PCBs in hair	Yang et al., 2015
Gold nano- particles	Capture probe	Thiol capped dsDNA	Target protein	qPCR	Detection of 3,4,3,Yang et al., 2014
Magnetic microparticles	Capture probe	Barcoded aptamers	Cytochrome c	SPR	Detection of cytochrome c	Loo et al., 2017
Gold nano– particles	Capture probe	Oligonucleotide sequences	miRNA	Fluorescence	Determination of different levels of miRNA levels from cancer cells	Lund et al., 2006
Nanowires	Label	Oligonucleotide sequences	Target DNA	Optical reflectance microscopy	Identification of specific DNA hybridization events	Lyberopoulou et al., 2015
Nanowires	Capture probe	Oligonucleotide sequences	Target DNA	Fluorescence	Detection and quantification of DNA	Lee et al., 2007

#### Nanobots/Nanorobots

Nanorobotics exhibits wide applications in the field of medicine. It is used for the early diagnosis and monitoring of various diseases and targeted drug delivery (Shetty et al., 2013). Nanobots on denitrification can be consumed as toothpaste and or mouthwash for covering all the subgingival surface areas. These are also used for trapping toxic organic materials into colorless and harmless vapors. They can perform functions at the cellular level when injected into patients. The examples of nanobots may include ultra–sensitive bio–chips. Because of their splendid technological characteristics, they can be utilized as biobarcoding.

#### Nanotubes

Nanotubes are generally made up of carbon molecules. These have a cylindrical shape, and are used in electronics, material sciences, and nanotechnology. These exhibits astounding unique strength and electrical properties. When bound with gold nanoparticles, they are used to develop special type of biosensors that can sense various types of cancer (Mishra, 2019). A wide variety of materials such as semiconductors, metals, and polymers have been developed using nanotubes technology (Escrig et al., 2008). Literature shows that magnetic nanotubes have been industriously investigated. However, barcode–types of nanostructures were less able to get attention, in spite of customizing their multisegmented nanotube structure utilizing their functionalization with various molecules such as DNA and proteins. Furthermore, it is hypothesized that these can be useful in the development of barcode–type magnetic nanostructures, molecules separation, and magnetic and biological sensors (Stoermer et al., 2006).

#### Quantum Dots/Nanocrystals

Quantum dots (QDs)/nanocrystals can be defined as fluorescent crystalline inorganic nanomaterials. These novel dots emit fluorescent light whose color or wavelength depends upon the size of dots (Shah et al., 2015). These are usually less than 1 μm in size. It has been reported that 10 nm nanocrystals seem sound as semi–conductors exhibiting nanopores situated between the crystals. Surface of these nanopores could adsorb proteins through the addition of silica molecules. These hydroxyapatite nanoparticles can also be utilized for the detection of bone defects (Jackson et al., 2017). These dots specifically produce a spectrum of colors when embedded into specific microbeads in excitation state. This makes QDs very handy for image–guided surgery, molecular diagnostics, and genotype determination. These dots can be operated as diagnostics as well as therapeutics in conjugation with different diagnostic techniques. For instance, when QDs get conjugated with fluorescence microscopy, help to observe living cells and labeling of various cancer markers (Jackson et al., 2017). Other applications of the quantum dot may include multiplexed detection and viral diagnostics (Maralla and Bharathi, 2019). It has been reported that QDs have several expedient features when compared to fluorescent dyes, for instance, (i) the dots can be excited by the same narrow band sources; (ii) these are less prone to photobleaching; (iii) these have symmetric and narrow emission spectra; (iv) higher encodings capacity; and (v) better signal to noise (Rauf et al., 2010). Researchers have developed and characterized a new modern type of barcoded magnetic bead using conjugated QDs through a layer–by–layer assembly approach. These magnetic based barcodes can be spotted on the basis of various spectral responses from different QDs which are assembled on the bead surface via biological self–assembly of quantum dot–biotin and quantum dot–streptavidin conjugates. These can be exploited to use the free biotin binding site on the biologically assembled code for the utilization of these quantum barcodes in the designing of a multiplex model qualitative immunoassay (Rauf et al., 2010).

#### Nanowires

Nanowires are deemed as tiny channels derived from metal oxides, silicon, and or carbon nanotubes. Nanowires may exhibit very low amplitude electrical current and have been proved to be sensitive to minute changes in electrical currents (Reimhult and Höök, 2015). These are used as probes when antibodies get attached to their surfaces. The interaction of these antibodies with the target biomolecules results in conformational changes in provision of electrical signals from these nanowires for interpretations. The attachment of nanowires with antibodies can be used to form sensitive devices that can detect various types of diseases (Reimhult and Höök, 2015). It has been reported that silicon nanowires can be extensively used to detect biomarkers of prostate cancer. Another research has shown that zinc and silicon nanowires can detect DNA as the binding of these p–type nanowires to negatively charged poly–anionic macromolecule enhanced electrical conductance. Furthermore, these DNA biosensors can be able to probe all types of mutations in cancer like diseases (Lyberopoulou et al., 2015). Barcode arrangements of nanowires are considered as a special case because of their specific arraying, and multiple functionality with enhanced properties when compared to their single–component counterparts. For example, Co–Cu barcoded nanowires have been developed with high magnetoresistance for the detection, separation, and transport of cells (Lee et al., 2007).

**FIGURE 1:**
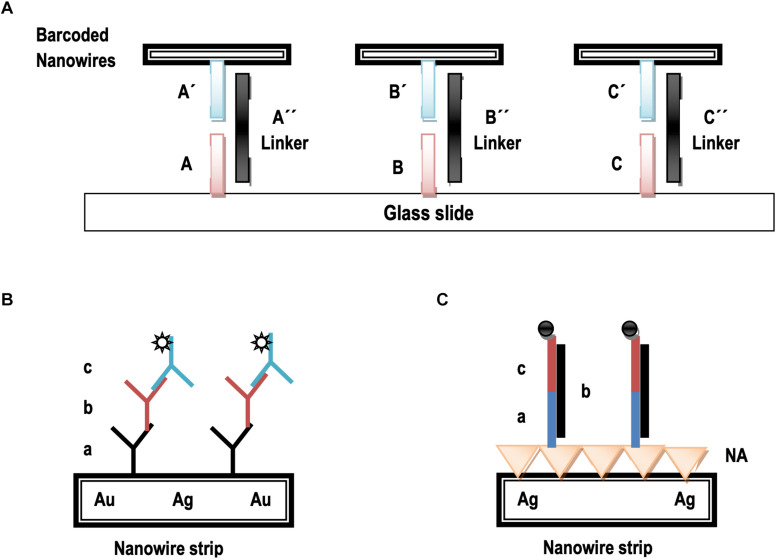
An illustration of nonfluorescent bioassay using bio–barcoded nanowires. **(A)** DNA strands labeled A–C are likely to be not complementary to the respective primed counterparts **(A′–C′)**. The strands double prime (″) labeled are likely to link the respective sequences to their primed counterparts through complementary DNA hybridization. **(B)** The striped particles are derivatized using capture antibody **(a)**, analyte **(b)**, and the fluorescent labeled detection antibody addition **(c)**. **(C)** The striped particles are derivatized through NeutrAvidin (NA), a biotinylated capture sequence’s reaction **(a)**, a special phase analyte **(b)**, and fluorescent labeled oligonucleotides for detection **(c)**.

Barcoded nanowires are a promising alternative to fluorescent tags in some applications. These particles can be used as coded substrates for biosensing, analog to DNA microarray chips, alternative to tags for detection and identification, and substitute to nanocrystals or fluorescent molecules (Nicewarner–Peña et al., 2003). Barcoded nanowires may be used as tags for the detection and identification of DNA hybridizations. A glass slide spotted with multiple capture sequences of DNA selective to the 3′ or 5′ region of the target sequences is utilized in this approach. Labeled double prime (") and target sequences are supposed to bind to the surface through hybridization to these specific capture strands. These can then be detected by hybridization-driven assembly of bio-barcoded nanowires which generally carry a third strand of DNA that is complementary to the 5′ or 3′ (or) region of the specific targeted sequences (Nicewarner–Peña et al., 2003). The wires bound to each of the spots on the surface can be counted and identified by optical reflectance microscopy which ultimately presents the amount and identification of targeted molecules or specimens present in the samples. This method is substantially simple with no requirement of fluorescence tags. A use of selective nanoparticles attachment will deliver a multiplexed analysis at broader scale (Figure 1A; Nicewarner–Peña et al., 2003).

It has been investigated that nanowires serve as identifying markers as well as detectable substrates for binding (Wang, 2008). Figure 1B shows the usage of barcoded nanowires as substrates for DNA and antigen detection. It demonstrates that a standard sandwich immunoassay using nanowires surface for the detection of antigens or proteins. An analogous detection of DNA detection using nanowires has been shown in Figure 1C, where nanowires serve as the substrates for probes attachment.

#### Bio-Chips and Microarrays

Chip-based nanotechnology has emerged as a new epitome for total chemical analysis systems. Chips can be silicon or a glass-based simple device that may have many processes for DNA analysis. A chip usually consists of channels in which biomolecules flow to biosensors. It consists of heat-based sensors for temperature, fluorescence detectors, fluidic channels, and electrophoretic chambers which are designed with microfabricates for nano-sized DNA analysis. Nanotechnology-based chips may measure biomolecules such as DNA digestion, DNA isolation, and DNA analysis (Jain, 2003). These novel innovations make biological and chemical information easier for healthcare and molecular diagnostics. Proteins based nanochips and nanofluidic arrays are examples of these devices. It may be postulated that detection of microorganisms, development of medicines, systems biology, and personalized medicines can be the unique possibilities of these techniques (Bahadorimehr et al., 2010).

#### Magnetic Nanoparticles

Magnetic nanoparticles (MNPs) are a type of iron nanoparticles. Their size may range in 15–20 nm. These particles can be constructed from glycidyl methacrylate with embodiment in copolymer beads (Jackson et al., 2017). Super MNPs have been described for calcium-sensing and tracking of cells. MNPs conjugated with magnetic resonance imaging (MRI) disclose small and undetectable metastasis of lymph nodes. Conjugation of super paramagnetic iron oxide enhances MRI for cerebral ischemic lesions. It has been documented that dextran coated MNPs may enhance MRI for intracranial visualization of tumors (Jackson et al., 2017). It has been reported that DNA bio-barcoded assays use oligonucleotide-modified magnetic gold nanoparticles for amplification and for separation of a targeted protein from the samples (Jackson et al., 2017). The theory of the bio-barcoded assay is special and presents a potential alternative source to the PCR technique.

#### Gold Nanoparticles

Gold nanoparticles manifest small pieces of DNA and gold particles within range of 13 nm in diameter. These can be particularly used as labels for the development of various types of sensors (Mieszawska et al., 2013). It has been observed that oligonucleotide-functionalized gold nanoparticles (AuNPs) have advantages in terms of performance, selectivity, and sensitivity over conventional probes in a wide variety of bio-detection protocols. The bio-barcode assays are generally based on AuNPs functionalization with a number of strands of oligonucleotides strands (assumed as barcodes) and a corresponding recognition agent which can be antibody in terms of protein detection, and a small segment of the barcoded strand in case of nucleic acids detection (Stoeva et al., 2006). Nanoprobes of magnetic gold nanoparticles when functionalized with locked nucleic acid and bio-barcode DNA, are used for the sensitive detection of miRNA.

#### Nanoshells

Literature reports show that nanoshells are mainly used in the delivery of chemotherapeutic agents to treat tumors. In its mechanism, drugs and polymer complexes are embedded in nanoshells and then injected into the body where they get accumulated near to tumors for their action. Nanoshells may melt by absorbing a certain infrared frequency from infrared rays resulting in site-specific drug release from polymer complex (Loo et al., 2004). The use of metal nanoshell-coated microbeads instead of uncoated microbeads reported a potential increase in analytical sensitivity for the detection of genetic targets. The assay process has been assumed to be very reliable, simple, and fast (Chapin and Doyle, 2011). It makes it advantageous as an ideal approach for ultra-sensitivity and high throughput multiplexed biosensing applications in clinical diagnosis (Chen et al., 2013), and for the detection of various organic targets including biological targets (Chan et al., 2019), such as peptides, pathogens, genomic and proteomic targets, nucleic acid sequences, amino acid sequences, carbohydrates, and lipids.

#### Nanopores

Data shows that nanopores can be implemented to sequence a complete codon in the DNA strand. It is ultimately an ultra-fast DNA sequencing. Detection and characterization of simple molecules by nanopores show a novel method for the interpretation of information directly from linear polymers (Soni and Meller, 2007). Specific individual DNA and proteins can be detected using solid-state nanopores through engineering of programmed binding sites at the centre of double-stranded long DNA. The DNA is likely to act as a “carrier” which selectively drives out proteins through the nanopores (Li et al., 2015; Bell and Keyser, 2016).

#### Nanobiosensors

Nanobiosensors are presumed as the nanosensors used for diagnosis of diseases through detection of specific biological and chemical entities (Escrig et al., 2008). These sensors can detect specific cells and peculiar body areas. Nanobiosensors are usually constructed of two entities; (1) biological entity which is used as sampling and (2) physical entity for sampling toward result production and transduction. These may differentiate between normal cells and cancerous cells by detecting specific biomolecules released by such cells. Genetic defects or mutations can be probed earlier by recognizing peculiar DNA using these sensors. These sensors can be of many types such as electrochemical nano biosensors, optical biosensor, carbon nanotube biosensors, nanowire biosensors, viral nanosensors, ion channel switch biosensors, and quartz nanobalance DNA sensors (Escrig et al., 2008). Current research is trying to amplify weak peptides biomarkers coated on nanomaterials which can be released into the bloodstream by specific proteases. These proteases are produced by cancerous cells and released into the urine where these can be ultimately detected (Escrig et al., 2008). Researchers have developed a very highly sensitive and selective biosensor for the detection of microRNAs (miRNAs) utilizing bio-barcode DNA assay with catalytic hairpin assembly via multiple probes (Dong et al., 2015). A new development of bio-barcoded biosensors for the detection of DNA in poor biological samples has been proclaimed using nanomagnetic beads (Trévisan et al., 2010).

## DNA Barcoding and Nanotechnology

### DNA Barcoding of Bacteria

In current scenario, PCR methods are going to be replaced by nanotechnology usage for nucleic acid detection. Scientific literature shows that fluorescence probes such as fluorescent metal ions (Zhang et al., 2013), nanoparticles (Zanoli et al., 2012), metal complexes, and bio-barcoded DNA are likely to be sole bio-detection methods which represent PCR-like sensitivity for both nucleic acid and proteins exempting the need of enzymatic amplification. Thus, the use of nanomaterials has opened a new eon in bio-analytical technology by introducing “Fluorescence bio-barcode DNA assay.” This assay involves the use of nanoparticles of metals and semiconductors exhibiting exclusive electronic, optical, catalytic and magnetic properties (Saha et al., 2012; Li and Antonietti, 2013).

It has been reported that fluorescent, super magnetic and metallic nanostructures are of critical importance for bioimaging and detection of infectious microorganisms, viruses, and bacteria in biological samples. Literature shows that “fluorescent bio-barcode DNA assay” has been used to probe the *Salmonella enteritidis* genes (Yin et al., 2012). This assay was based on two types of nanoparticles: (1) Gold nanoparticles, and (2) MNPs. *S. enteritidis* has been recognized as an infectious bacterium that causes diarrhea, fever, and abdominal cramps after transmitted in body. This technique can detect even trace amounts of pathogens with high sensitivity and proficiency to save human lives. Similarly, gold, silver, and nickel barcoded nanowires have been reported to be used for the detection of bacterial antigens that have been supposed to be life-threatening.

Conjugated polymer nanoparticles (CPNs) are used in molecular imaging or cell imaging. These are very critical in drug discovery, gene profiling, and clinical diagnosis. Their potential use is based on the self-assembly of CPNs and bacteria which lead to multicolor emissions. Various color-barcoded micro particles are prepared by mixing of *Escherichia coli* and CPNs together. These multicolored particles exhibit low toxicity toward cells and can be widely applicable for optical barcoding and cell imaging (Khan et al., 2019). Nowadays, barcoded nanoparticles (BNPs) are used to detect specific gene sequences in bacteria. In this method, target DNA is sandwiched between nanoparticle and microparticle probes containing barcode DNA. Thus, barcode DNA can be detected by using a universal probe (Amini et al., 2016).

**FIGURE 2:**
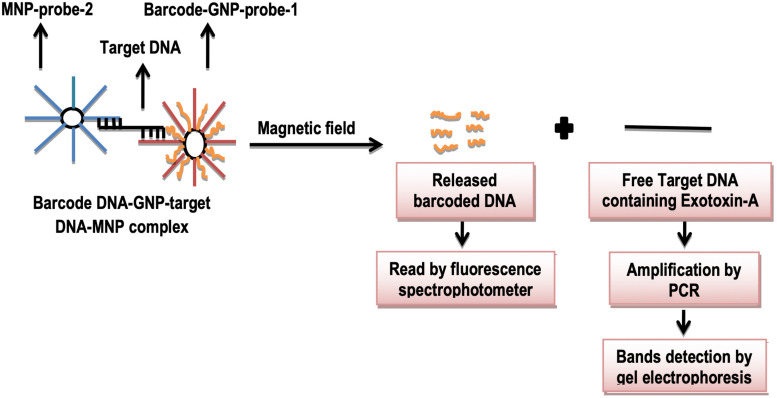
Schematic illustration of DNA barcoding assay.

Amini along with colleagues in 2016 detected exotoxin-A by fluorescence bio-barcode DNA assay based on magnetic and gold nanoparticles as shown in Figure 2. *Pseudomonas aeruginosa* is a gram-negative aerobic bacterium. It produces exotoxin-A (ETA) which presumes to be a very toxic substance (Tok et al., 2006). If this bacterium enters the human body, then it releases exotoxin-A which may lead to stomach disturbance, vomiting, and even mortality (Feng et al., 2012). For this purpose, thiol-capped alternative oligonucleotide sequences are taken as probe-1 and probe-2. DNA barcode is made as thiol-capped. Probe-2 forms complex on the surface of MNPs, while probe-1 and barcode DNA form a complex with gold nanoparticles (GNPs). After this, the target DNA of *P. aeruginosa* is allowed to co-hybridize with both probes to form a sandwich structure. The target DNA of *P. aeruginosa* is then separated by applying a magnetic field, and free barcode DNA is detected by fluorescence spectrophotometer. From target DNA, the gene for exotoxin-A is amplified by PCR, and resultant bands are observed by gel electrophoresis. However, this technique requires less time with unfortunate less sensitivity when compared to conventional and classical methods of detection (Amini et al., 2016).

Traditional detection methods of bacterial infections are time-consuming, complex with lack of detection of pooled samples of pathogens. To counter these mentioned challenges, “Barcoded quantum dot assay” can be demonstrated as effective alternative tool. This essay utilizes magnetic and non-magnetic micro- and nanoparticles providing a most suitable platform for barcoded assay (Zhao et al., 2015). The nanoparticles act as carriers for molecules for the detection of a targeted component. The capturing molecules spotted on nanoparticles and the targeted component of the bacteria must have affinity of specific binding. The capturing molecules are nucleic acids and antibodies in the form of genes or oligonucleotides. Barcode assay for the detection of bacteria is generally based on labeled oligonucleotides because antibodies may pose a cross reactivity and difficult availability. For the detection of microorganisms in a multiplex analysis, and identification of individual or exclusive species, QDs are considered to be highly preferable (Zahavy et al., 2010).

**FIGURE 3:**
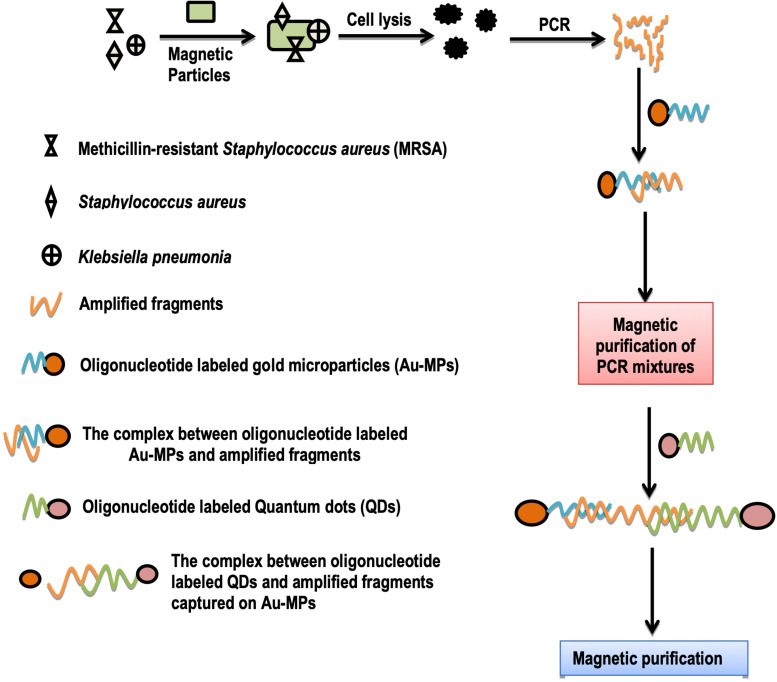
Schematic illustration of detection of three bacterial species by barcode-quantum dot assay.

Researchers used “barcode-quantum dot (Fluorescent nanoparticles) assay” for the detection of three bacterial pathogens: *Methicillin-resistant Staphylococcus aureus* (MRSA), *Staphylococcus aureus*, and *Klebsiella pneumonia* (Nam et al., 2005; Figure 3). They took a mixture of these bacteria and added this mixture to magnetic particles, which non-specifically capture these bacteria. After this, these bacteria were lysed, and resulting bacterial lysates were also amplified by multiplex PCR. Gold magnetic particles (oligonucleotides barcode labeled) were then added, which magnetically purify the individual bacteria by complementary binding to their oligonucleotide barcodes. In the end, QDs (oligonucleotides barcode labeled) were allowed to bind with their complementary binding sites on Au-MPs. Final products were magnetically purified which represented the separate genes for three specific bacterial species. This method has been proven very sensitive which can detect even minute concentrations of bacteria (Nam et al., 2005).

### DNA Barcoding and Detection of Cytokines

**FIGURE 4:**
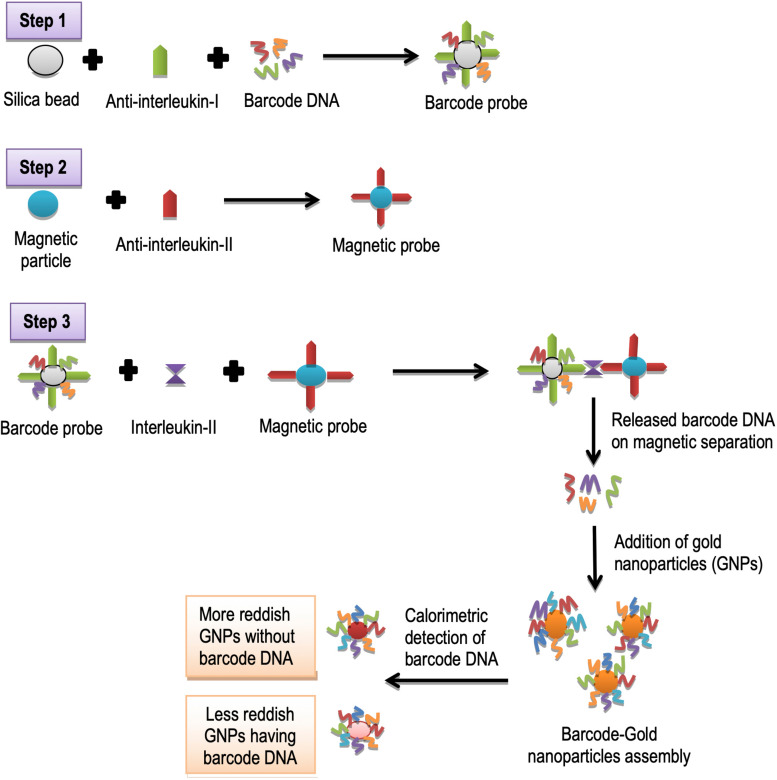
Schematic illustration of detection of interleukin-II using DNA barcoding and nanotechnology.

Cytokines are small proteins that aid cell signaling in immune responses. Interleukin-2 (IL-2) is a cytokine protein, secreted during inflammation, and immune responses in humans. It shows local interactions between white blood cells (Zhao et al., 2015). Advanced method for the detection of these biological targets such as protein (IL-2), and nucleic acid is known as “bio-barcode amplification assay.” This assay is based on porous particles of silica and gold nano particles-based on calorimetric DNA detection schemes (Figure 2). The porous particles enable the loading of millions of barcodes of DNA through amplification principle (Zahavy et al., 2010). Previously, some researchers detected cytokines proteins using this method. This method encompasses three steps (Nam et al., 2005); (1) The barcode probe is prepared by immobilizing monoclonal antibodies (IL-2) to amino-functionalized porous silica microparticles. Then amino-functionalized barcode DNA complements are added to modify silica particles; (2) magnetic probe preparation is carried out when the monoclonal antibody for IL-2 is immobilized to Amino-functionalized magnetic particles; (3) barcode DNA quantification is carried out when this magnetic probe is allowed to form a complex with a barcode probe in the presence of IL-2. In abutting step, using magnetic separations, many barcode DNAs are released. These released barcode DNAs are then captured on gold nanoparticles. By spotting particles on the TLC plate and detection of barcode DNA is carried out using colorimeter. In interpretation, nanoparticles without barcode DNA appear more reddish as compared to the nanoparticles with barcode DNA (Nam et al., 2005; Figure 4).

It has been observed that classical methods for the detection need many tedious experimental steps such as microarray-based immobilization of oligonucleotide on a glass chip, silver enhancement of immobilized gold nanoparticles on a chip, light scattering measurement, and a quantification step. Experimental requirements can be minimized by using the colorimetric bio-barcode method with the potential use of nanotechnology (Nam et al., 2005).

### DNA Barcoding and Analysis of Neurotransmitters

Data shows that “nanoparticle based bio-barcode DNA assay” presents a highly sensitive detection of nucleic acids and proteins (Cao et al., 2010). This assay includes two types of particles: magnetizable spheres having modified a functional group with an affinity for the target, and nanoparticles with a second modified group that possesses an affinity for the same target, and as well as for an oligonucleotide barcode DNA that is likely to be a reporter for the target. The nanoparticle-based bio-barcode DNA assay proclaims the use of gold nanoparticles as a key feature. This warrants a simultaneous loading of detection probes/antibodies, a huge quantity of barcoded DNA per single gold nanoparticle, and high sensitivity by avoiding the complicated preparation of antibody – DNA conjugate as required in immuno-PCR (Duan et al., 2010).

**FIGURE 5:**
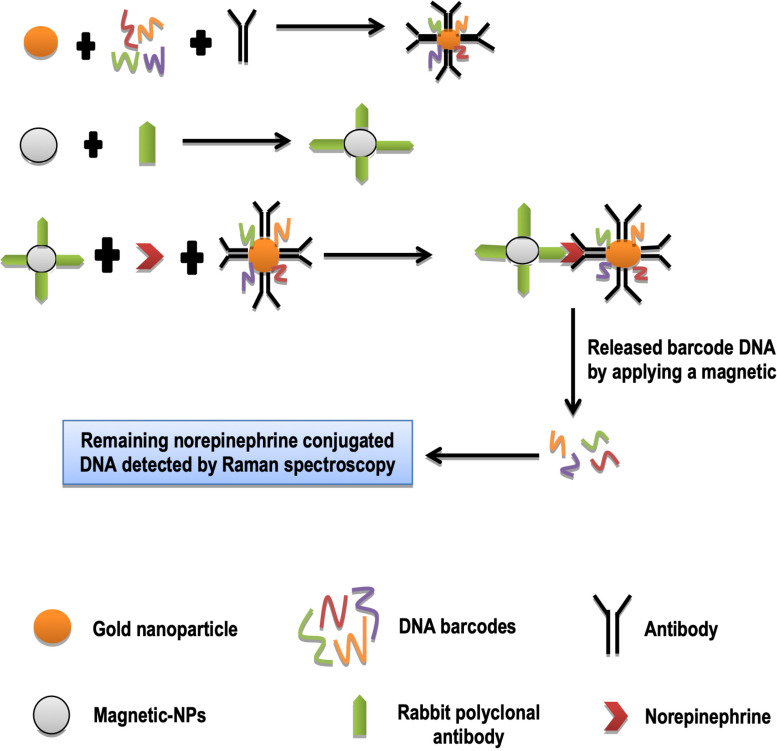
Quantification of norepinephrine.

Neurotransmitters are sort of chemical messengers that carry signals from neurons. Norepinephrine is a neurotransmitter that belongs to a class of catecholamines. It controls motivation, alertness, heartbeat, gastrointestinal activity, and blood pressure (Shankaran and Miura, 2007). “Nanoparticles based bio-barcode assay” is a very advanced technique. It is used to analyze nucleic acids and proteins. The number of neurotransmitters can also be determined by using a nanoparticles-based bio-barcode technique. Rabbit’s norepinephrine polyclonal antibodies were mixed with magnetizable polystyrene beads in a PCR tube and incubated at 37°C for 24 h. Gold nanoparticles were treated with antibodies and oligonucleotides as bio-barcodes. The norepinephrine made a sandwich structure between the rabbit polyclonal antibodies, and the antibodies of gold nanoparticles. This sandwich structure was separated magnetically. Resultant norepinephrine (NE) barcodes DNA was analyzed by surface-enhanced Raman spectroscopy (Hee et al., 2016; Figure 5). This tool is rapid, and has high throughput screening for analyzing neurotransmitters as compared to conventional methods. In recent years, diagnosis and treatment of neurological disorders are the most challenging issues. In nifty words, using DNA barcoding and nanotechnology, it will be possible to diagnose all neurological disorders by detecting neurotransmitters which are presumed to be involved in these disorders (Hee et al., 2016).

### DNA Barcoding and Assessment of Sensitivity of Anticancer Drugs

**FIGURE 6:**
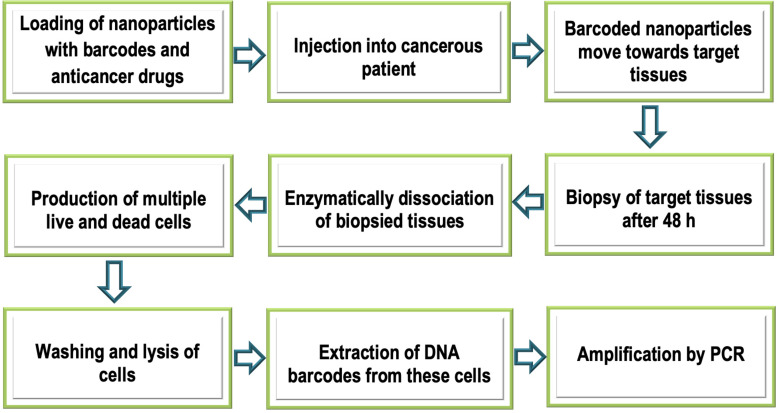
Schematic illustration of analysis of tumor sensitivity of anticancer drugs.

Anticancer drugs perform their function only if being sensitive to cancer disease. Assessment of the sensitivity of anticancer drugs is recognized very critical prior their recommendation to the patients (Yaari et al., 2016). BNPs are also used to probe the sensitivity of tumors toward anticancer drugs. For this purpose, researchers showed loading of nanoparticles with anticancer drugs (Yaari et al., 2016). Each nanoparticle was loaded with at least 15 barcodes. These BNPs were then injected intravenously to the cancer patients. BNPs were found to move toward target cancer tissue inside the body of the patients pursuing therapeutic activity. Approximately 48 h later, a biopsy was taken from the tumor tissues. Biopsied tissues were dissociated enzymatically generating multiple cells, and each cell was sorted by FACS (fluorescence-activated cell sorting) according to their viability. Live and dead cells were washed, lysed, and DNA barcodes from them were extracted. These barcodes were amplified by real-time PCR. In presumption determination, barcodes found in the dead cells belong to active drugs, while those of living cells belong to inactive drugs. On the basis of the sensitivity of anticancer drugs, thus barcodes could be suggested for treatment and to enhance treatment target’s sensitivity (Yaari et al., 2016; Figure 6). Silica nanoparticles bound to proteins surfaces can be used as nano bio-chips. This provides a lot of potential for detecting cancerous cells. Protein enzymes or antibodies are immobilized on a glass slide. This chip is probed with a sample that binds to a relevant protein on the chip, and is analyzed for detection. Proteins on chips can be used to differentiate normal cells from cancerous cells (Stoermer et al., 2006).

Conventional methods for the assessment of drugs sensitivity may include quantitative scoring approach, analytical methods, and analytical instrumentation which desire many experimental steps to assess the sensitivity of anticancer drugs. However, nano-based DNA barcoded chips are very sensitive technique to predict the therapeutic efficacy of anticancer drugs. In the future, work should be done to check the sensitivity of all anticancer drugs by using DNA barcoding and nanotechnology. Specific sensors using nanotechnology will measure the sensitivity of anticancer drugs by detecting tumor-specific DNA barcodes (Stoermer et al., 2006).

## Biobarcoding and Nanotechnology

### Aptamers and Nanoparticles Integration for Barcoding

**FIGURE 7:**
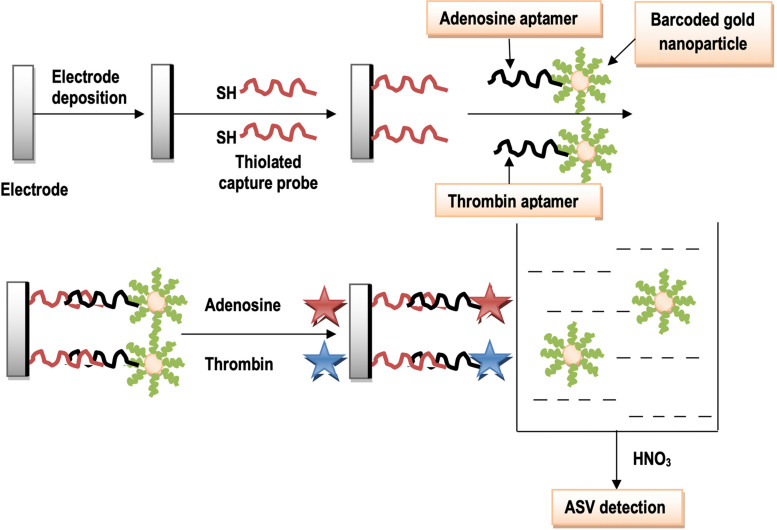
Schematic illustration of the formation of sensing interface using aptamers with AuNPs.

Aptamers are defined as receptor molecules made up of single- and/or double-stranded oligonucleotides. These are obtained from large libraries by an *in vitro* sequential process of systematic evolution of ligands by exponential enrichment (SELEX; Li et al., 2010). Aptamers have clinched an attention to be important molecular tools in diagnostics because of their affinity, inherent selectivity, flexibility, and stability (Li et al., 2010). Aptamer-based biosensors (aptasensors) possess unprecedented advantages compared with biosensors using natural receptors such as antibodies and enzymes (Li et al., 2010). Li et al. (2010), introduced an electrochemical sensing strategy for a quick and simultaneous detection of thrombin and adenosine-based aptamers as switching structures. A gold electrode was used as a sensor which was modified with two types of thiolated capture probes complementary to the linker DNA. The linker DNA holds either a thrombin aptamer or an adenosine aptamer. The capture probes have the ability to hybridize with the corresponding linker DNA. The AuNPs possess two types of bio-barcode DNA; (a) one is complementary to the linker DNA (as reporter), while the other is not (signal), but is tagged with a different type of metal sulfide nanoparticles. Thus, a “sandwich-type” sensing surface has been developed fabricated with adenosine and thrombin. The aptamer parts (adenosine and thrombin) bind with their targets to form the quite complex structures. This results in the release of bio-barcoded AuNPs into solution. The concentration of adenosine and thrombin is supposed to be proportional to the signal of either metal ion, while the measurement of metal sulfide nanoparticles comes up by anodic stripping voltammetry (ASV; Figure 7; Li et al., 2010).

### Barcoding and Detection of Allergens

Allergy is caused by plant pollens or some foodstuff which contain allergenic proteins that cause allergic reactions (Hochwallner et al., 2014). “Mass barcode-based mass spectrometry (MS) signal amplification” has been introduced as a very advanced technique used in tissue imaging (Yan et al., 2013), immunoassay (Lee et al., 2008), and DNA assays (Qiu et al., 2008). In this technique, small tagged molecules (mass barcodes) are attached to micro or nanoparticles, and are targeted to large biomolecules. After this, they can easily be detected by MS that results in an amplified signal of the targets. The combination of mass barcodes and magnetic beads has been proved to be a very effective for MS detection of large biomolecules, e.g., allergens (Nam et al., 2015).

**FIGURE 8:**
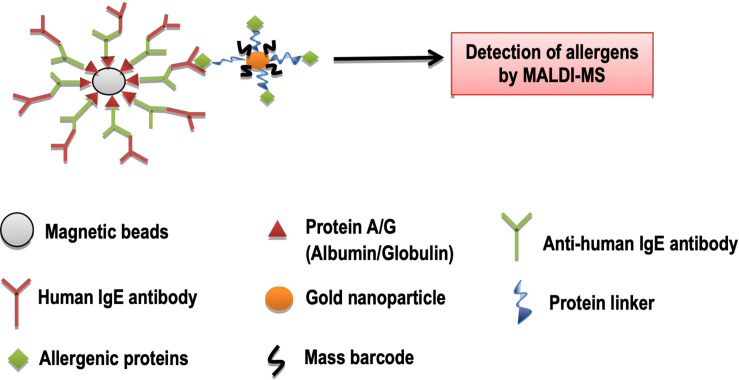
Detection of allergens by MALDI-MS. Legends: MALDI, Matrix-assisted laser desorption/ionization; MS, Mass spectrometry.

In mass barcode signal amplification, mass barcode amplification is used with the combination of commercial protein-coated magnetic beads, which diagnose multiplex allergy components with MS detection. The molecules of polyethylene glycol are chosen as a mass barcode that have different chain lengths. These mass barcodes are placed on biological chips and or plates which act as surface. Then these barcodes are made to join with gold nanoparticles, which further get attachment with commercially available magnetic beads linked A/G allergenic proteins to design gold nanoparticles probes. These probes are attached to anti-human IgE antibodies that make a sandwich structure. On detection by MS, it indicates the presence of specific IgE antibodies (Zhong et al., 2016; Figure 8). Traditional immunoassay methods for the detection of allergens are time-consuming and they are not very highly sensitive, but recent mass barcodes amplification in combination with magnetic beads proves to be highly sensitive in detecting allergens. In the future, this technology would be helpful for the treatment of different allergies (Zhong et al., 2016).

### Barcoding and Detection of Viruses

Barcoded-nanotechnology is used to detect viruses using “barcode lateral flow immune-assay.” A barcode lateral flow immunoassay based on magnetic nanoparticle is used to optimize antibody affinity for a specific antigen. To increase sensitivity and broaden the applications of lateral flow immunoassay, modifications have been made by controlling the cut-off level and insertion of MNPs (Zvereva et al., 2015; Orlov et al., 2016). In this way, this assay can be used to diagnose hormones, cardiac and cancer biomarkers, and to detect plant diseases (Wang L. et al., 2014). Research has shown that the cut-off level of sensitive barcode lateral flow assay with magnetic nanoparticle for the detection of potato viruses. In the initial stage, monoclonal (3G4 and 1A5), and polyclonal mouse antibodies (pAb) specific to PVX (potato virus X) are developed. Then, immune complexes are formed between monoclonal and polyclonal antibodies, potato virus and secondary anti-mouse antibodies (IgG) on the chip surface. Consequently, MNPs make conjugates with immune complexes. Transmission electron microscopy is brought in use to visualize the immune complexes with MNPs (magnetic nanoparticles) followed by barcode lateral flow assay for the detection of potato viruses. The lateral flow test strips are dipped in a solution containing immune complexes with and without MNPs (Panferov et al., 2016). Nanowire field-effect transistors are also used to detect viral pathogens. Viral antibodies modify binding and unbinding characteristics of nanowire arrays. It helps in the real-time detection of selective viruses (Shah et al., 2015).

**FIGURE 9:**
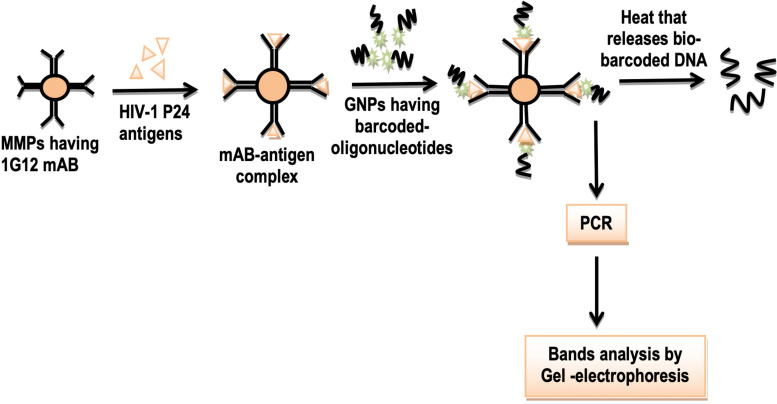
Schematic illustration of detection of HIV p24 antigen using barcoding and nanotechnology. Legends: MMPs, *Matrix metalloproteinases*; 1G12 mAB, 1G12 monocolonal antibody. GNPs, Gold nanoparticles; and PCR, polymerase chain reaction.

Human immunodeficiency virus (HIV) p24 is a major disease-causing antigen of the human immunodeficiency virus (HIV-1; Fiebig et al., 2003). In the past, this antigen was detected by the ELISA technique, but the ELISA process is time consuming with low sensitivity. Currently, BCA assay based on gold nanoparticles and magnetic microparticles has been introduced to detect this antigen in less time and high sensitivity. Researchers have shown that this assay can be used to detect HIV p24 antigen. In that process, microplates or magnetic microplates are coated with a 1G12 monoclonal antibody (Tang et al., 2007). Then, HIV p24 antigens are made to be treated to form a complex with these monoclonal antibodies. Gold nanoparticles are modified by the attachment of barcoded oligonucleotides (DNA). Then these gold nanoparticles are allowed to form a sandwich complex with these antigens. This complex is heated to release bio-barcode DNA and followed to get amplified by PCR. The resulting bands are separated through gel electrophoresis (Figure 9). In biomedical research, work has been started to diagnose viral diseases using this potential technology (Dong et al., 2012).

### Barcoding and Evaluation of Ligand-Receptor Affinity

**FIGURE 10:**
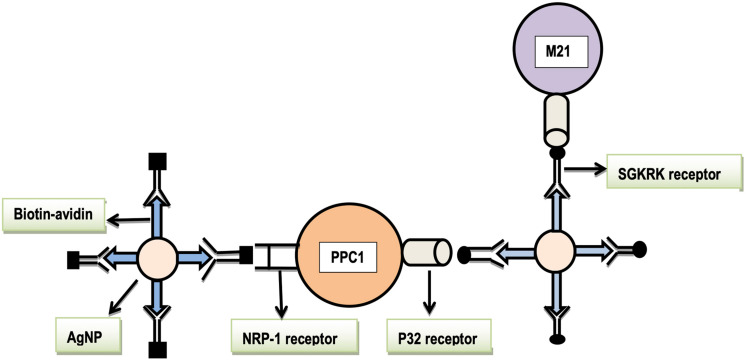
The affinity of barcoded AgNPs to their respective tumor cell receptors. Legends: NRP-1, neuropilin-1, SGKRK, a peptide name, AgNPs, silver nanoparticles, PPC1, cancer cell line type, and M21, melanoma cancer cell line type.

Nanoparticles are delivered to cells and tissues on the basis of the affinity of ligand bounded nanoparticles to their specific receptors on cells or tissues. Willmore and colleagues demonstrated that barcoded silver nanoparticles (AgNPs) can be used to determine the binding affinity of the ligand with tumor cell receptors (Agemy et al., 2013; Chaudhary et al., 2014). They made AgNPs isotopically, i.e., by mixing silver and palladium isotopes and coated these nanoparticles with Neutravidins (NA). Two types of barcoded ligands were used; the first one is RPARPAR (tumor penetrating peptide) and the second is SGKRK (tumor homing peptide). In their demonstration, these barcoded ligands were bounded to biotin by amino hexanoic acid as intermediate. Then this biotin part of barcoded peptide ligands attached to the NeutrAvidin part of AgNPs. They made three types of barcoded nanoparticles, (I) RPARPAR biotin AgNPs, (II) SGKRK biotin AgNPs, and (III) Control biotin AgNPs (with no peptide ligand linkage). Two types of cancer cells were used, PPC-1 prostate cancer cell and M21 melanoma cells. These cells were incubated with these two barcoded nanoparticles and control biotin nanoparticles (Agemy et al., 2013; Chaudhary et al., 2014). Binding affinity between tumor cell receptors and barcoded nanoparticles was assessed by plasma mass spectrometry. The SGKRK barcoded nanoparticles demonstrated an affinity to bind with p32 receptors on M21 melanoma cells. The RPARPAR barcoded nanoparticles showed an affinity to bind with NRP-1 receptors on PPC-1 (prostate cancer cells; Fogal et al., 2008; Teesalu et al., 2009; Agemy et al., 2011; Figure 10 and Table 2). P32 protein is a mitochondrial chaperone, while NRP-1 is the neutrophilic-1 receptor. Both are overexpressed on the surface of tumor cells. It is presumed that barcoding and nanotechnology will be used to check the affinity of anticancer drugs to tumor cell receptors so that scientists would be able to treat cancer at best (Agemy et al., 2013; Chaudhary et al., 2014).

**TABLE 2:** The affinity of barcoded AgNPs to their respective tumor cell receptor.

Barcoded AgNPs	Specific receptors	Targeted tumor cells
RPARPAR-AgNPs	NRP-1 and p32	PPC-1 prostate cancer cell
SGKRK-AgNPs	Only P32	M21 melanoma cells
Control biotin AgNPs (with no barcode)	Have no affinity with any receptors	–

### Generation of Multicolored Bio-Barcodes

The effect of changing colored barcodes and errors in barcodes with different nanoparticles can be determined *in vitro*. The purpose of this technology is to ensure the possible number of colored barcodes using sequential loading protocol (Agemy et al., 2013). For this purpose, varied numbers of nanoparticle loaded vesicles as a colored barcode are produced using a protocol named “Sequential loading protocol.” This protocol involves a cell population that is sequentially loaded with nanoparticles carrying different emission wavelengths. Every cell in the population generates its own barcode. Then the effect of changing colors of barcode and misreading in cellular code on encoding the cells is observed (Agemy et al., 2013).

At present, this is a recent technology that has been set forth to use nanoparticle colored barcode for tracking and identification of cells. Nano based generation of multicolored barcodes has been raised as a rapid, and highly sensitive technique as compared to the other conventional methods. It is anticipated that multicolored barcodes will be used for multiplex detection in coming years (Agemy et al., 2013).

### Generation of Optical Bio-Barcodes

It has been reported that various optical barcodes can be generated using nanotechnology. These barcodes manifest their use in multiplex detection (Wang G. et al., 2014). These optical barcodes can be manipulated by mixing (polystyrene-co-maleic anhydride; PSMA) microsphere near-infrared emitting QDs, and Fe_3_O_4_ containing super-paramagnetic nanoparticles by membrane emulsification technique. As a result, various bifunctional optical barcodes are produced. In emulsification technique, optical barcodes can be encoded by the “single-wavelength” encoding model that can be used to detect tumor or other biomarkers (Rees et al., 2016). This is deduced as a very sensitive method of detection compared to traditional ELISA, magnetic microspheres technology, and fluorescence-encoded microsphere technology (Wang G. et al., 2014). It is speculated that special sensors can be designed that can quickly encode these optical barcodes.

### Generation of Digital Bio-Barcodes

Literature proclaims that digital bio-barcodes simultaneously detect multiple biomolecular analytes with more precision. Currently, it is possible to generate digital barcodes by “Laser-induced breakdown spectroscopy” (LIBS) in which nanomaterials are well used (Leng et al., 2016). In principle, this technique generates digital barcodes with high sensitivity as compared to other conventional digital methods. LIBS is a method that converts analog barcodes into a digital form (He et al., 2016). By LIBS technology, analysis of industrial products, drugs or medicines, and monitoring of environment can be carried out efficiently. LIBS based polystyrene microspheres are prepared by using layer-by-layer self-assembly method, carrying some surface modifications. These microspheres are encoded with nanoparticles. Subsequently, some binding groups are coated on the surface of these microspheres that help in the binding of these microspheres to target analytes (e.g., ssDNA; Lin et al., 2013). The analytes are labeled with QDs as fluorescent tags. Fluorescent tags and nanoparticles are stimulated by a pulse laser, which shows colored emissions to detect target analytes. On the basis of colored coding emissions, a map of digital barcodes is generated. These digital barcodes can be further used in multiplex detection. In the future, work will be conducted to develop such biosensors which can quickly encode these digital barcodes with high sensitivity and precision (Lin et al., 2013).

### Diagnosis of Different Pathogenic Organisms

The predominant conventional methods to identify the pathogens rely upon clinical monitoring approaches. These methods are based upon culturing the microorganisms which are contemplated as laborious, expensive, and time-consuming (Khiyami et al., 2014; Sautter et al., 2015). Another drawback of the conventional methods is that these do not support to manage a large number of clinical samples. In contrast, the “nano-barcode diagnostic systems” are more accurate and reliable tools for the detection of pathogenic microorganisms. Nano-barcode diagnostic system is a system used to analyze DNA, RNA, proteins, mycotoxins, bacteria, and viruses. In this assay, oligonucleotide associated magnetic gold nanoparticles (AuMNPs) are used. This assay is very effective and more proficient than PCR technology as it has high sensitivity to diagnose the pathogens in a short span of time. However, the nanobarcode diagnosis of fungi, algae, protozoa, and some pollutants in food and agriculture is an under-phase area, and it requires more work for further exploration (Khiyami et al., 2014; Sautter et al., 2015).

### Diagnosis of Diseases by Quantum Dot Barcode Technology

Quantum dots are synthesized by semi-conductor materials. Their size is about 10 nm, and more than one antigen can be detected through QD barcode technology (Giri et al., 2011). QDs emit various colors by absorbing a specific wavelength. Like metallic nanoparticles, QDs are utilized in nano-enhanced imaging for environmental monitoring, molecular diagnostics, and related treatment (Giri et al., 2011). “Bio-barcode quantum dot technology” has been introduced to diagnose diseases by detecting disease-causing antigens (Gao et al., 2011; Rai and Ingle, 2012). This technology involves QD barcodes which can be synthesized by using porous silica beads, swelling process, polymerization, and by fluidic-flow focusing approach. In this process, antigens form complex with QD barcodes and give an inferring signal (Giri et al., 2011). For the detection of one antigen, a specific buffer solution is prepared and antigen detecting probes are dispersed in this solution. These probes are fluorescent/radioactive labeled. QD based bio-barcodes are added in it and antigen will form a complex with barcodes, and show fluorescence which can be read out by the optical device. This type of assay in which only one antigen or anyone molecule is detected is called single-plexed QD barcode assay (Jennings et al., 2008). While in multiplexed QD barcode assay, multiple analytes are detected by forming many QD barcodes. These QD barcode assays can be used to diagnose multiple range of diseases. It can be predicted for the future that this tool will be utilized for clinical validation of important diseases (Kim et al., 2016).

## Conclusion

The progressive development of diagnostic methods for the analysis of pathogens, allergens, diseases, and their treatment is a hallmark task in the modern era. The avalanche approaches in nanoscience reveal how biological data can be acquired quickly, easily, and inexpensively, then interpreted, thus enormously increase the possibilities of wonderful achievements. These may also lead to a move in diagnosis, and therapy with new outcomes. DNA barcoding and biobarcoding combined with potential nanotechnology fulfill technological advancement. Contribution of nanobarcoding toward multiplex detection, diagnosis, and treatment of diseases, is highly promising and rapidly growing.

## Future Perspectives

In the future, this innovative approach will be nifty and/or worthwhile for the detection of fungi, algae, protozoa, and various range of pollutants. It will also be agile for the development of advanced nano-biosensors in life sciences. Furthermore, nanotechnology interplay with bio-barcodes will lead for nanoscale products which can then be used with entailed biological systems. The new dimension of nanotechnology research may focus on recognizing cells and their constituents, and individual genes of impaired functions and their self-repair. It can easily be predicted that this field would witness a same exponential growth as the other two technological field such as information technology in the year of 1960s and biotechnology in 1980s witnessed earlier.

## Author Contributions

SM and SA drafted the DNA and nanotechnology part, while MI and MK wrote the biobarcoding part of the manuscript. SCO and SA conceived the idea, reviewed, and edited the manuscript. All authors read and approved the final version of manuscript.

## Conflict of Interest

The authors declare that the research was conducted in the absence of any commercial or financial relationships that could be construed as a potential conflict of interest.

## Abbreviations

PVX: potato virus X
dsDNA: double stranded DNA
ssDNA: single stranded DNA
CRP: C-reactive protein
IL: interleukin
PSA: prostate specific antigen
TAP: triazophous
qPCR: real-time PCR
MS: mass spectrometry
SRP: surface plasmon resonance
miRNA: micro ribonucleic acid
and PCB: polychlorinated biphenyls.

## References

[B1] Agemy L. Friedmann-Morvinski D. Kotamraju V. R. Roth L. Sugahara K. N. Girard O. M. (2011). Targeted nanoparticle enhanced proapoptotic peptide as potential therapy for glioblastoma. *Proc. Natl. Acad. Sci. U.S.A.* 108 17450–17455. 10.1073/pnas.1114518108 21969599 PMC3198371

[B2] Agemy L. Kotamraju V. R. Friedmann-Morvinski D. Sharma S. Sugahara K. N. Ruoslahti E. (2013). Proapoptotic peptide-mediated cancer therapy targeted to cell surface p32. *Mol. Ther.* 21 2195–2204. 10.1038/mt.2013.191 23959073 PMC3863797

[B3] Amini B. Kamali M. Salouti M. Yaghmaei P. (2016). Fluorescence bio-barcode DNA assay based on gold and magnetic nanoparticles for detection of Exotoxin A gene sequence. *Biosens. Bioelectron.* 92 679–686. 10.1016/j.bios.2016.10.030 27838203

[B4] Bahadorimehr A. Yunas J. Majlis B. Y. (2010). “Low cost fabrication of microfluidic microchannels for Lab-On-a-Chip applications,” in *Proceedings of the 2010 International Conference on Electronic Devices, Systems and Applications*, (Kuala Lumpur: IEEE), 242–244.

[B5] Bao Y. P. Wei T.-F. Lefebvre P. A. An H. He L. Kunkel G. T. (2006). Detection of protein analytes via nanoparticle-based bio bar code technology. *Anal. Chem.* 78 2055–2059. 10.1021/ac051798d 16536446

[B6] Bao Y. P. Wei T. F. Lefebvre P. A. An H. He L. Kunkel G. T. (2006). Detection of protein analytes via nanoparticle-based bio bar code technology. *Anal. Chem.* 78 2055–2059. 10.1021/ac051798d 16536446

[B7] Bell N. A. Keyser U. F. (2016). Digitally encoded DNA nanostructures for multiplexed, single-molecule protein sensing with nanopores. *Nat. Nanotechnol.* 11:645 10.1038/nnano.2016.50 27043197

[B8] Broto M. Galve R. Marco M. P. (2017). Sandwich NP-based biobarcode assay for quantification C-reactive protein in plasma samples. *Anal. Chim. Acta* 992 112–118. 10.1016/j.aca.2017.09.007 29054144

[B9] Cao C. Dhumpa R. Bang D. D. Ghavifekr Z. Høgberg J. Wolff A. (2010). Detection of avian influenza virus by fluorescent DNA barcode-based immunoassay with sensitivity comparable to PCR. *Analyst* 135 337–342. 10.1039/b916821b 20098768

[B10] Chan W. Leo Y. T. Chen K. (2019). *U.S. Patent Application No. 14/768,051.*

[B11] Chapin S. C. Doyle P. S. (2011). Ultrasensitive multiplexed microRNA quantification on encoded gel microparticles using rolling circle amplification. *Anal. Chem.* 83 7179–7185. 10.1021/ac201618k 21812442 PMC3173568

[B12] Chaudhary B. Khaled Y. S. Ammori B. J. Elkord E. (2014). Neuropilin 1: function and therapeutic potential in cancer. *Cancer Immunol. Immunother.* 63 81–99. 10.1007/s00262-013-1500-0 24263240 PMC11028473

[B13] Chen K. Chou L. Y. Song F. Chan W. C. (2013). Fabrication of metal nanoshell quantum-dot barcodes for biomolecular detection. *Nano Today* 8 228–234. 10.1016/j.nantod.2013.04.009

[B14] Coissac E. Hollingsworth P. M. Lavergne S. Taberlet P. (2016). From barcodes to genomes: extending the concept of DNA barcoding. *Mol. Ecol.* 25 1423–1428. 10.1111/mec.13549 26821259

[B15] Cowan R. S. Fay M. F. (2012). Challenges in the DNA barcoding of plant material. *Methods Mol. Biol.* 862 23–33. 10.1007/978-1-61779-609-8_3 22419486

[B16] Dong H. Liu J. Zhu H. Ou C.-Y. Xing W. Qiu M. (2012). Two types of nanoparticle-based bio-barcode amplification assays to detect HIV-1 p24 antigen. *Virol. J.* 9:180 10.1186/1743-422x-9-180 PMC3496641 22935171

[B17] Dong H. Meng X. Dai W. Cao Y. Lu H. Zhou S. (2015). Highly sensitive and selective microRNA detection based on DNA-bio-bar-code and enzyme-assisted strand cycle exponential signal amplification. *Anal. Chem.* 87 4334–4340. 10.1021/acs.analchem.5b00029 25830473

[B18] Duan R. Zhou X. Xing D. (2010). Electrochemiluminescence biobarcode method based on cysteamine- gold nanoparticle conjugates. *Anal. Chem.* 82 3099–3103. 10.1021/ac100018z 20297795

[B19] Escrig J. Bachmann J. Jing J. Daub M. Altbir D. Nielsch K. (2008). Crossover between two different magnetization reversal modes in arrays of iron oxide nanotubes. *Phys. Rev. B* 77 214421.

[B20] Feng X. Yang G. Liu L. Lv F. Yang Q. Wang S. (2012). A convenient preparation of multi-spectral microparticles by bacteria-mediated assemblies of conjugated polymer nanoparticles for cell imaging and barcoding. *Adv. Mater.* 24 637–641. 10.1002/adma.201102026 21932281

[B21] Ferri G. Alù M. Corradini B. Angot A. Beduschi G. (2008). Land plants identification in forensic botany: multigene barcoding approach. *Foren. Sci. Int. Genet. Suppl. Ser.* 1 593–595. 10.1016/j.fsigss.2007.10.023

[B22] Fiebig E. W. Wright D. J. Rawal B. D. Garrett P. E. Schumacher R. T. Peddada L. (2003). Dynamics of HIV viremia and antibody seroconversion in plasma donors: implications for diagnosis and staging of primary HIV infection. *Aids* 17 1871–1879. 10.1097/00002030-200309050-00005 12960819

[B23] Fogal V. Zhang L. Krajewski S. Ruoslahti E. (2008). Mitochondrial/cell-surface protein p32/gC1qR as a molecular target in tumor cells and tumor stroma. *Cancer Res.* 68 7210–7218. 10.1158/0008-5472.can-07-6752 18757437 PMC2562323

[B24] Gao Y. Stanford W. L. Chan W. C. (2011). Quantum-dot-encoded microbeads for multiplexed genetic detection of non-amplified DNA samples. *Small* 7 137–146. 10.1002/smll.201000909 21110335

[B25] Ghosh S. Majumder P. Mandi S. S. (2011). Species-specific AFLP markers for identification of *Zingiber officinale*, Z. *montanumand Z. zerumbet* (Zingiberaceae). *Genet. Mol. Res.* 10 218–229. 10.4238/vol10-1gmr1154 21341214

[B26] Giri S. Sykes E. A. Jennings T. L. Chan W. C. (2011). Rapid screening of genetic biomarkers of infectious agents using quantum dot barcodes. *ACS Nano* 5 1580–1587. 10.1021/nn102873w 21355538

[B27] Goluch E. D. Stoeva S. I. Lee J.-S. Shaikh K. A. Mirkin C. A. Liu C. (2009). A microfluidic detection system based upon a surface immobilized biobarcode assay. *Biosens. Bioelectron.* 24 2397–2403. 10.1016/j.bios.2008.12.017 19157846 PMC2749686

[B28] Grodzinski P. Silver M. Molnar L. K. (2014). Nanotechnology for cancer diagnostics: promises and challenges. *Exp. Rev. Mol. Diagn.* 6 307–318. 10.1586/14737159.6.3.307 16706735

[B29] He Q. Liu Y. He Y. Zhu L. Zhang Y. Shen Z. (2016). Digital barcodes of suspension array using laser induced breakdown spectroscopy. *Sci. Rep.* 6:36511. 10.1038/srep36511 PMC5093434 27808270

[B30] Hebert P. D. Gregory T. R. (2005). The promise of DNA barcoding for taxonomy. *Syst. Biol.* 54 852–859. 10.1080/10635150500354886 16243770

[B31] Hee J. An K.-J. Lee Choi J.-W. (2016). Gold nanoparticles-based barcode analysis for detection of Norepinephrine. *J. Biomed. Nanotechnol.* 12 357–365. 10.1166/jbn.2016.2185 27305769

[B32] Hochwallner H. Schulmeister U. Swoboda I. Spitzauer S. Valenta R. (2014). Cow’s milk allergy: from allergens to new forms of diagnosis, therapy and prevention. *Methods* 66 22–33. 10.1016/j.ymeth.2013.08.005 23954566 PMC3969108

[B33] Jackson T. C. Patani B. O. Ekpa D. E. (2017). Nanotechnology in diagnosis: a review. *Adv. Nanopart.* 6:93 10.4236/anp.2017.63008

[B34] Jain K. K. (2003). Nanodiagnostics: application of nanotechnology in molecular diagnostics. *Exp. Rev. Mol. Diagn.* 3 153–161. 10.1586/14737159.3.2.153 12647993

[B35] Jeevanandam J. Barhoum A. Chan Y. S. Dufresne A. Danquah M. K. (2018). Review on nanoparticles and nanostructured materials: history, sources, toxicity and regulations. *Beilstein J. Nanotechnol.* 9 1050–1074. 10.3762/bjnano.9.98 29719757 PMC5905289

[B36] Jennings T. Rahman K. Fournier-Bidoz S. Chan W. (2008). Effects of microbead surface chemistry on DNA loading and hybridization efficiency. *Anal. Chem.* 80 2849–2856. 10.1021/ac7026035 18307362

[B37] Khan I. Saeed K. Khan I. (2019). Nanoparticles: properties, applications and toxicities. *Arab. J. Chem.* 12 908–931. 10.1016/j.arabjc.2017.05.011

[B38] Khiyami M. A. Almoammar H. Awad Y. M. Alghuthaymi M. A. Abd-Elsalam K. A. (2014). Plant pathogen nanodiagnostic techniques: forthcoming changes? *Biotechnol. Biotechnol. Equip.* 28 775–785. 10.1080/13102818.2014.960739 26740775 PMC4684063

[B39] Kim J. Biondi M. J. Feld J. J. Chan W. C. (2016). Clinical validation of quantum dot barcode diagnostic technology. *ACS Nano* 10 4742–4753. 10.1021/acsnano.6b01254 27035744

[B40] Lebonah D. Dileep A. Chandrasekhar K. Sreevani S. Sreedevi B. Pramoda Kumari J. (2014). DNA barcoding on bacteria: a review. *Adv. Biol.* 2014 1–9.

[B41] Lee J. H. Wu J. H. Liu H. L. Cho J. U. Cho M. K. An B. H. (2007). Iron–gold barcode nanowires. *Angew. Chem. Int. Ed.* 46 3663–3667. 10.1002/anie.200605136 17407120

[B42] Lee J. R. Lee J. Kim S. K. Kim K. P. Park H. S. Yeo W. S. (2008). Mass spectrometry signal amplification method for attomolar detection of antigens using small-molecule-tagged gold microparticles. *Angew. Chem. Int. Ed.* 47 9518–9521. 10.1002/anie.200803893 18972477

[B43] Leng Y. Wu W. Li L. Lin K. Sun K. Chen X. (2016). Magnetic/fluorescent barcodes based on cadmium-free near-infrared-emitting quantum dots for multiplexed detection. *Adv. Funct. Mater.* 26 7581–7589. 10.1002/adfm.201602900

[B44] Li T. Liu L. Li Y. Xie J. Wu H. C. (2015). A universal strategy for aptamer-based nanopore sensing through host–guest interactions inside α-hemolysin. *Angew. Chem. Int. Ed.* 54 7568–7571. 10.1002/anie.201502047 25966821

[B45] Li X. Xia J. Li W. Zhang S. (2010). Multianalyte electrochemical biosensor based on aptamer-and nanoparticle-integrated bio-barcode amplification. *Chem. Asian J.* 5 294–300. 10.1002/asia.200900217 20013991

[B46] Li X. H. Antonietti M. (2013). Metal nanoparticles at mesoporous N-doped carbons and carbon nitrides: functional Mott–Schottky heterojunctions for catalysis. *Chem. Soc. Rev.* 42 6593–6604. 10.1039/c3cs60067j 23765224

[B47] Lim J. Kim S.-Y. Kim S. Eo H.-S. Kim C.-B. Paek W. K. (2009). BioBarcode: a general DNA barcoding database and server platform for Asian biodiversity resources. *BMC Genomics* 10:S8 10.1186/1471-2164-10-S3-S8 PMC2788395 19958506

[B48] Lin Q. Niu G. Wang Q. Yu Q. Duan Y. (2013). Combined laser-induced breakdown with Raman spectroscopy: historical technology development and recent applications. *Appl. Spectro. Rev.* 48 487–508. 10.1080/05704928.2012.751028

[B49] Liu D. Huang Y. Wang S. Liu K. Chen M. Xiong Y. (2015). A modified lateral flow immunoassay for the detection of trace aflatoxin M1 based on immunomagnetic nanobeads with different antibody concentrations. *Food Control* 51 218–224. 10.1016/j.foodcont.2014.11.036

[B50] Loo C. Lin A. Hirsch L. Lee M.-H. Barton J. Halas N. (2004). Nanoshell-enabled photonics-based imaging and therapy of cancer. *Technol. Cancer Res. Treat.* 3 33–40. 10.1177/153303460400300104 14750891

[B51] Loo J. F. C. Yang C. Tsang H. L. Lau P. M. Yong K. T. Ho H. P. (2017). An Aptamer Bio-barCode (ABC) assay using SPR, RNase H, and probes with RNA and gold-nanorods for anti-cancer drug screening. *Analyst* 142 3579–3587. 10.1039/c7an01026e 28852760

[B52] Lund K. Williams B. Ke Y. Liu Y. Yan H. (2006). DNA nanotechnology: a rapidly evolving field. *Curr. Nanosci.* 2 113–122. 10.2174/157341306776875811

[B53] Lyberopoulou A. Efstathopoulos E. Gazouli M. (2015). Nanodiagnostic and nanotherapeutic molecular platforms for cancer management. *J. Cancer Res. Updates* 4 153–162. 10.6000/1929-2279.2015.04.04.3

[B54] Lyle S. N. Lourtioz J.-M. Lahmani M. Dupas-Haeberlin C. Hesto P. (2015). *Nanosciences and Nanotechnology: Evolution or Revolution?.* Berlin: Springer.

[B55] Maralla S. Bharathi D. (2017). New modalities for drug delivery in the treatment of chronic diseases through Nanosize drug delivery systems. *International Journal of Pharmacy and Life Sciences* 8, 5407–5411.

[B56] Mieszawska A. J. Mulder W. J. Fayad Z. A. Cormode D. P. (2013). Multifunctional gold nanoparticles for diagnosis and therapy of disease. *Mol. Pharmaceut.* 10 831–847. 10.1021/mp3005885 PMC3593826 23360440

[B57] Mishra A. K. (2019). *Application of Nanotechnology in Diagnosis, Drug Dissolution, Drug Discovery, and Drug Carrier, Nanobiotechnology in Bioformulations.* Berlin: Springer, 449–475.

[B58] Nam J. Yoo M. Yeo W.-S. (2015). Measurement of prostate-specific antigen level as a biomarker for breast cancer by using mass signal amplification. *BioChip J.* 9 124–129. 10.1007/s13206-015-9205-4

[B59] Nam J.-M. Wise A. R. Groves J. T. (2005). Colorimetric bio-barcode amplification assay for cytokines. *Anal. Chem.* 77 6985–6988. 10.1021/ac0513764 16255599

[B60] Nicewarner-Peña S. R. Carado A. J. Shale K. E. Keating C. D. (2003). Barcoded metal nanowires: optical reflectivity and patterned fluorescence. *J. Phys. Chem. B* 107 7360–7367. 10.1021/jp034139i

[B61] Nithaniyal S. Newmaster S. G. Ragupathy S. Krishnamoorthy D. Vassou S. L. Parani M. (2014). DNA barcode authentication of wood samples of threatened and commercial timber trees within the tropical dry evergreen forest of India. *PLoS One* 9:e107669 10.1371/journal.pone.0107669 PMC4178033 25259794

[B62] Ochs H. D. Smith C. E. Puck J. M. (2006). *Primary Immunodeficiency Diseases: a Molecular & Cellular Approach.* Oxford: Oxford University Press.

[B63] Orlov A. V. Bragina V. A. Nikitin M. P. Nikitin P. I. (2016). Rapid dry-reagent immunomagnetic biosensing platform based on volumetric detection of nanoparticles on 3D structures. *Biosens. Bioelectron.* 79 423–429. 10.1016/j.bios.2015.12.049 26741530

[B64] Panferov V. G. Safenkova I. V. Zherdev A. V. Dzantiev B. B. (2016). Setting up the cut-off level of a sensitive barcode lateral flow assay with magnetic nanoparticles. *Talanta* 164 69–76. 10.1016/j.talanta.2016.11.025 28107991

[B65] Peng S. Derrien T. L. Cui J. Xu C. Luo D. (2012). From cells to DNA materials. *Mater. Today* 15 190–194. 10.1016/s1369-7021(12)70089-5

[B66] Qiu F. Jiang D. Ding Y. Zhu J. (2008). Monolayer-barcoded nanoparticles for on-chip DNA hybridization assay. *Angew. Chem.* 120 5087–5090. 10.1002/ange.200800435 18506872

[B67] Rai M. Ingle A. (2012). Role of nanotechnology in agriculture with special reference to management of insect pests. *Appl. Microbiol. Biotechnol.* 94 287–293. 10.1007/s00253-012-3969-4 22388570

[B68] Rajpoot A. Kumar V. P. Bahuguna A. Kumar D. (2016). DNA barcoding and traditional taxonomy: an integrative approach. *Int. J. Curr. Res.* 8, 42025–42031.

[B69] Rauf S. Glidle A. Cooper J. M. (2010). Application of quantum dot barcodes prepared using biological self-assembly to multiplexed immunoassays. *Chem. Commun.* 46 2814–2816. 10.1039/b927149j 20369192

[B70] Rees P. Rowan M. Brown J. W. Wills Summers H. (2016). An analysis of the practicalities of multi-color nanoparticle cellular bar-coding. *Combinat. Chem. High Throughput Screen.* 19 362–369. 10.2174/1386207319666160408150649 27055751

[B71] Reimhult E. Höök F. (2015). Design of surface modifications for nanoscale sensor applications. *Sensors* 15 1635–1675. 10.3390/s150101635 25594599 PMC4327096

[B72] Roh S. Chung T. Lee B. (2011). Overview of the characteristics of micro-and nano-structured surface plasmon resonance sensors. *Sensors* 11 1565–1588. 10.3390/s110201565 22319369 PMC3274020

[B73] Saha K. Agasti S. S. Kim C. Li X. Rotello V. M. (2012). Gold nanoparticles in chemical and biological sensing. *Chem. Rev.* 112 2739–2779. 10.1021/cr2001178 22295941 PMC4102386

[B74] Sautter V. Toplis M. Wiens R. Cousin A. Fabre C. Gasnault O. (2015). In situ evidence for continental crust on early Mars. *Nat. Geosci.* 8 605–609.

[B75] Shah A. Jani M. Venkatesh C. Thukral N. Patel S. (2015). Nano-technology in dentistry: a review. *J. Adv. Med. Dent. Sci. Res.* 3:60.

[B76] Shankaran D. R. Miura N. (2007). Recent progress and challenges in nanotechnology for biomedical applications: an insight into the analysis of neurotransmitters. *Recent Patents Nanotechnol.* 1 210–223. 10.2174/187221007782360484 19076034

[B77] Shetty N. J. Swati P. David K. (2013). Nanorobots: future in dentistry. *Saudi Dent. J.* 25 49–52. 10.1016/j.sdentj.2012.12.002 23960556 PMC3723292

[B78] Smith M. A. Rodriguez J. J. Whitfield J. B. Deans A. R. Janzen D. H. Hallwachs W. (2008). Extreme diversity of tropical parasitoid wasps exposed by iterative integration of natural history, DNA barcoding, morphology, and collections. *Proc. Natl. Acad. Sci. U.S.A.* 105 12359–12364. 10.1073/pnas.0805319105 18716001 PMC2518452

[B79] Soni G. V. Meller A. (2007). Progress toward ultrafast DNA sequencing using solid-state nanopores. *Clin. Chem.* 53 1996–2001. 10.1373/clinchem.2007.091231 17890440

[B80] Stark W. J. Stoessel P. R. Wohlleben W. Hafner A. (2015). Industrial applications of nanoparticles. *Chem. Soc. Rev.* 44 5793–5805. 25669838 10.1039/c4cs00362d

[B81] Stoermer R. L. Cederquist K. B. McFarland S. K. Sha M. Y. Penn S. G. Keating C. D. (2006). Coupling molecular beacons to barcoded metal nanowires for multiplexed, sealed chamber DNA bioassays. *J. Am. Chem. Soc.* 128 16892–16903. 10.1021/ja0658261 17177440 PMC2849162

[B82] Stoeva S. I. Lee J. S. Thaxton C. S. Mirkin C. A. (2006). Multiplexed DNA detection with biobarcoded nanoparticle probes. *Angew. Chem. Int. Ed.* 45 3303–3306. 10.1002/anie.200600124 16602131

[B83] Tallury P. Malhotra A. Byrne L. M. Santra S. (2010). Nanobioimaging and sensing of infectious diseases. *Adv. Drug Deliv. Rev.* 62 424–437. 10.1016/j.addr.2009.11.014 19931579 PMC7103339

[B84] Tang S. Zhao J. Storhoff J. J. Norris P. J. Little R. F. Yarchoan R. (2007). Nanoparticle-based biobarcode amplification assay (BCA) for sensitive and early detection of human immunodeficiency type 1 capsid (p24) antigen. *JAIDS J. Acq. Immune Defic. Syndrom.* 46 231–237. 10.1097/qai.0b013e31814a554b 17693896

[B85] Teesalu T. Sugahara K. N. Kotamraju V. R. Ruoslahti E. (2009). C-end rule peptides mediate neuropilin-1-dependent cell, vascular, and tissue penetration. *Proc. Natl. Acad. Sci. U.S.A.* 106 16157–16162. 10.1073/pnas.0908201106 19805273 PMC2752543

[B86] Thaxton C. S. Elghanian R. Thomas A. D. Stoeva S. I. Lee J.-S. Smith N. D. (2009). Nanoparticle-based bio-barcode assay redefines “undetectable” PSA and biochemical recurrence after radical prostatectomy. *Proc. Natl. Acad. Sci. U.S.A.* 106 18437–18442. 10.1073/pnas.0904719106 19841273 PMC2773980

[B87] Thaxton C. S. Hill H. D. Georganopoulou D. G. Stoeva S. I. (2005). A bio-bar-code assay based upon dithiothreitol-induced oligonucleotide release. *Anal. Chem.* 77 8174–8178. 10.1021/ac0514265 16351173

[B88] Tok J. B. H. Chuang F. Kao M. C. Rose K. A. Pannu S. S. Sha M. Y. (2006). Metallic striped nanowires as multiplexed immunoassay platforms for pathogen detection. *Angew. Chem. Int. Ed.* 45 6900–6904. 10.1002/anie.200601104 16888828

[B89] Trévisan M. Schawaller M. Quapil G. Souteyrand E. Mérieux Y. Cloarec J. P. (2010). Evanescent wave fluorescence biosensor combined with DNA bio-barcode assay for platelet genotyping. *Biosens. Bioelectron.* 26 1631–1637. 10.1016/j.bios.2010.08.038 20851593

[B90] Wang G. Leng Y. Guo H. Song S. Jiang Z. Yuan X. (2014). Efficient preparation of magnetic quantum dot barcodes. *J. Mater. Chem. B* 2 8310–8313. 10.1039/c4tb01672f 32262000

[B91] Wang J. (2008). Barcoded metal nanowires. *J. Mater. Chem.* 18 4017–4020. 10.1039/b803807d

[B92] Wang L. Cai J. Wang Y. Fang Q. Wang S. Cheng Q. (2014). A bare-eye-based lateral flow immunoassay based on the use of gold nanoparticles for simultaneous detection of three pesticides. *Microchim. Acta* 181 1565–1572. 10.1007/s00604-014-1247-0

[B93] Wang P. Tian C. Li X. Mao C. (2014). Assembly of barcode-like nucleic acid nanostructures. *Small* 10 3923–3926. 10.1002/smll.201400942 24978689

[B94] Wanunu M. (2012). Nanopores: a journey towards DNA sequencing. *Phys. Life Rev.* 9 125–158. 10.1016/j.plrev.2012.05.010 22658507 PMC3780799

[B95] Yaari Z. da Silva D. Zinger A. Goldman E. Kajal A. Tshuva R. (2016). Theranostic barcoded nanoparticles for personalized cancer medicine. *Nat. Commun.* 7:13325. 10.1038/ncomms13325 PMC5109543 27830705

[B96] Yan B. Kim S. T. Kim C. S. Saha K. Moyano D. F. Xing Y. (2013). Multiplexed imaging of nanoparticles in tissues using laser desorption/ionization mass spectrometry. *J. Am. Chem. Soc.* 135 12564–12567. 23931011 10.1021/ja406553f PMC3801209

[B97] Yang G. Zhuang H. Chen H. Ping X. Bu D. (2015). A gold nanoparticle based immunosorbent bio-barcode assay combined with real-time immuno-PCR for the detection of polychlorinated biphenyls. *Sens. Actuat. B Chem.* 214 152–158. 10.1016/j.snb.2015.02.128

[B98] Yang G. X. Zhuang H. S. Chen H. Y. Ping X. Y. Bu D. (2014). A sensitive immunosorbent bio-barcode assay based on real-time immuno-PCR for detecting 3, 4, 3′, 4′-tetrachlorobiphenyl. *Anal. Bioanal. Chem.* 406 1693–1700. 10.1007/s00216-013-7583-9 24458480

[B99] Yin H. Q. Jia M. X. Yang S. Wang S. Q. Zhang J. G. (2012). A nanoparticle-based bio-barcode assay for ultrasensitive detection of ricin toxin. *Toxicon* 59 12–16. 10.1016/j.toxicon.2011.10.003 22005297

[B100] Zahavy E. Heleg-Shabtai V. Zafrani Y. Marciano D. Yitzhaki S. (2010). 1; Application of fluorescent nanocrystals (q-dots) for the detection of pathogenic bacteria by flow-cytometry. *J. Fluoresc.* 20 389–399. 19826932 10.1007/s10895-009-0546-z

[B101] Zanoli L. M. D’Agata R. Spoto G. (2012). Functionalized gold nanoparticles for ultrasensitive DNA detection. *Anal. Bioanal. Chem.* 402 1759–1771. 10.1007/s00216-011-5318-3 21866403

[B102] Zhang C. Du P. Jiang Z. Jin M. Chen G. Cao X. (2018). A simple and sensitive competitive bio-barcode immunoassay for triazophos based on multi-modified gold nanoparticles and fluorescent signal amplification. *Anal. Chim. Acta* 999 123–131. 10.1016/j.aca.2017.10.032 29254562

[B103] Zhang Y. Zhao H. Wu Z. Xue Y. Zhang X. He Y. (2013). A novel graphene-DNA biosensor for selective detection of mercury ions. *Biosens. Bioelectron.* 48 180–187. 10.1016/j.bios.2013.04.013 23685314

[B104] Zhao Y. Cheng Y. Shang L. Wang J. Xie Z. Gu Z. (2015). Microfluidic synthesis of barcode particles for multiplex assays. *Small* 11 151–174. 10.1002/smll.201401600 25331055

[B105] Zhong X. Qiao L. Gasilova N. Liu B. (2016). Mass barcode signal amplification for multiplex allergy diagnosis by MALDI-MS. *Anal. Chem.* 88 6184–6189. 10.1021/acs.analchem.6b01142 27244120

[B106] Zhou Z. Li T. Huang H. Chen Y. Liu F. Huang C. (2014). A dual amplification strategy for DNA detection combining bio-barcode assay and metal-enhanced fluorescence modality. *Chem. Commun.* 50 13373–13376. 10.1039/c4cc05554c 25233044

[B107] Zinger L. Philippe H. (2016). Coalescing molecular evolution and DNA barcoding. *Mol. Ecol.* 25 1908–1910. 10.1111/mec.13639 27169389

[B108] Zvereva E. A. Byzova N. A. Sveshnikov P. G. Zherdev A. V. (2015). Cut-off on demand: adjustment of the threshold level of an immunochromatographic assay for chloramphenicol. *Anal. Methods* 7 6378–6384. 10.1039/c5ay00835b

